# "The Hospital" Nursing Mirror

**Published:** 1900-01-20

**Authors:** 


"The Hospital" Nursing Mirror.

Being the Nursing Section of "The Hospital."

[Contributions for this Section of "The Hospital" should be addressed to the Editor, The Hospital, 28 & 29, Southampton Street, Strand, London, W.C., and should have the word "Nursing" plainly written in left-hand top corner of the envelope.]

### Notes on News from the Nursing World.

### LORD IVEAGH'S HOSPITAL.

It is a matter of inquiry why female nurses are not to accompany Lord Iveagh's Hospital to the front, with its hundred beds, thirty tents, twenty wagons, and eighty or a hundred mules and horses. The explanation is that no female nurses are allowed at the front. It is not a question of prejudice, but of regulations.

### THE EVICTED NURSES ASKED TO RETURN TO JOHANNESBURG.

A nurse who is now engaged at Maritzburg, and who mentions that General Buller has been very kind inquiring about the wounded, recites in a Bradford paper her experiences at the Johannesburg Hospital. When she first went there, directly after the Jameson raid, as one of the organisers, she found everything at sixes and sevens. She and her colleagues had "fearful hardships" to encounter. The place was "simply a hot-bed of fever," and two of the nurses died from the disease. However, the other nurses got through the trying times, and were all happy until the war broke out. Then fresh and worse misery commenced, and Dr. Mangold—who is now described as a German, and, it seems, is married to a niece of President Kruger—assumed control, and told the English nurses to clear out "in about twelve hours." This was after they had been told that they were to stay, and would be perfectly safe. In fact, they had actually begun to prepare for the wounded. The dreadful journey by Delagoa Bay to Durban has already been described; but the most interesting point in the nurse's letter is that she states Dr. Mangold himself was subsequently turned out "for his bad management," and the English nurses asked to come back. They did not, however, get the telegram, and only heard of the request being made when it was too late to comply with it.

### THE COMMERCIAL ELEMENT IN NURSING.

In an interview with the matron of the Royal Free Hospital, reported elsewhere, Miss Wedgwood was asked whether she considers that there is any foundation for the assertion that there is a tendency to make the commercial element too prominent in nursing. She thinks that the tendency exists, and she strongly deprecates it. Admitting that the principle of "so much work so much money" is very good up to a certain point, she affirms that the work is "utterly ruined if it is looked at from a purely commercial standpoint." There is no doubt that she is right. It was not for commercial ends that Miss Florence Nightingale became the pioneer of nursing as it is now known, and any members of the profession who put such considerations always first must inevitably lower rather than ennoble it.

### THE SCARCITY OF NURSES AT HOME.

Notwithstanding all that we hear about nurses volunteering for service at the front and on hospital ships, the supply at home is not just now by any means equal to the demand. On all sides we hear of the difficulty of obtaining them, and it is far from being confined to the metropolis. One explanation is that a considerable proportion of nurses themselves require nursing. At Leicester the other day one of the governors of the infirmary stated that there were no less than 15 nurses off duty owing to influenza. The other is, of course, that the sufferers from influenza continue to multiply so rapidly that if all nurses were in the enjoyment of the most robust health, their hands would be more than full. We are quite sure that, as a profession, they are no less willing to nurse influenza patients at home than wounded soldiers in South Africa.

### ST. GEORGE'S INFIRMARY, FULHAM ROAD.—RUMOURED RESIGNATIONS.

No defence from the parish authorities of St. George's, Hanover Square, in reference to the statements made by our correspondent respecting St. George's Infirmary, Fulham Road, has yet reached us; but we print a letter we have received, apparently sent us with the idea that the writer is doing the authorities a service. "Audi Alteram Partem," who does not say whether she is a probationer or not, makes curious admissions, notwithstanding her flat contradictions; and, quite unnecessarily, repeats that "the doctor and matron have the nurses and patients' welfare at heart," for it was never suggested that they had not. We are not surprised to learn from other sources that a commotion has been caused at the infirmary; that the question of wearing damp clothes has been discussed by the committee; and that several of the nurses are about to resign on account of over-work. We would remind our readers, however, that the matters in question have to do primarily with the welfare of the patients. Nurses can always obtain a hearing for their troubles, not so the patients in an institution under Poor Law rules; and when we find that the officials—for nurses are officials—express indignation at publicity being given to conditions within the workhouse walls which are admittedly far from what they should be, we are, to say the least of it, sorry. As a commentary on the protests of a few probationers against the publication of our correspondent's letter, we may mention that many of the nurses rejoice that the demand for searching reform has been made on grounds which, judging by the only answers forthcoming, are unanswerable.

### NURSING IN IRISH WORKHOUSES.

Lest anyone should think that finality has been reached in workhouse reform, or imagine that after all that has been done during the past 30 years to ameliorate the lot of the pauper sick we may now stay our hands, it is as well to be reminded, as we are by an article which appeared in the Irish *Figaro* a few weeks ago, on the realities of poorhouse life in Ireland, that wherever paupers are placed in authority over one another abuses are sure to arise, and that even the appointment of trained nurses gives no security against tyranny and favouritism if these nurses are allowed to delegate their authority or employ a pauper deputy in the nursing of the sick. It is distressing to think of, but the history of workhouse abuse, wherever and whenever it is inquired into, goes to show that it is not from the tyranny of officials, but from that of their fellow paupers that the sick in workhouses suffer the direst hardships. The tale told by "A Pauper" in the Irish *Figaro* brings up afresh, as now in full swing, the very abuses which were disclosed in the English infirmaries nearly forty years ago by Dr. Anstie, Dr. Rogers, Mr. Ernest Hart, and others. Notwithstanding the appointment of paid nurses, when the nurse's work is delegated to the wardsman everything goes wrong. "They," *i.e.*, the wardsmen, "curtail the patient's pint of milk to three noggins, and give the balance in mug-fulls to their favourites." The same with the eggs, which, when distributed by the wardsmen, go astray. History repeats itself. According to the *Irish Figaro* there seems to be a regular market for the sale of articles of diet, and especially of extras. The patient whose friends can provide him with a few halfpence a week is able to live in clover, while the friendless pauper is deprived of the comforts for which the guardians pay. It is the old tale over again, and only serves to show that now as before a workhouse infirmary, on which the master can turn the key, and from which he can exclude inquisitive inquirers, may easily become one of the darkest corners of the earth.

### THE MATRONS AT THE METROPOLITAN ASYLUMS BOARD HOSPITALS.

The Local Government Board have sanctioned the proposal of the Managers of the Metropolitan Asylums Board to assign to the office of matron at their infectious hospitals a salary of £100 a year, rising by £5 a year to a maximum of £150, with the usual emoluments; and have also approved of the amount of the proposed immediate increases in the salaries of the matrons already in the service.

### THE "LEICESTER" NURSES' HOME AT NORWICH.

There seems to be no limit to the munificence of the Earl of Leicester. At the quarterly meeting of the governors of the Norfolk and Norwich Hospital on Saturday it was announced that Lord Leicester had promised a further sum of £10,000, making in all £15,000, for the erection of a home for the nurses of the hospital. The new building is to be most appropriately called the Leicester Nurses' Home. That at a time like the present, when so many funds are appealing for support, Lord Leicester should have determined to provide the nurses of the institution, to which he has given a large fortune, with accommodation which will be second to none in the country must be particularly gratifying to them.

### THE MASTER OF THE ROLLS ON DISTRICT NURSING.

At the annual meeting of the Norfolk District and Cottage Nursing Federation, the Master of the Rolls was one of the speakers. In moving the adoption of the report he referred to the want of the greater facility for exchanging nurses from one district to another, and stated that another thing they wanted was "some kind of recognition by the County Council." As he pointed out, several County Councils offer scholarships for nurses, and he pleaded for the assistance of the Norfolk Council. Dr. Burton Fanning subsequently referred to the same subject, and strongly urged the Council to render help in training nurses. He declared that nobody would welcome a supply of nurses in the county more than the doctors, and said that "very often their position was one of absolute despair when they were called upon to take a case where a nurse was necessary, and one could not be obtained." It was not only, he added, the increased chance of recovery a nurse gave, "but it also very often required a nurse to prevent the spread of disease to the healthy members of the household." This is an aspect of affairs which is not always taken into account.

### MISS MARY KINGSLEY'S MISTAKE.

In her lecture at the Livingstone Exhibition reported last week, Miss Mary Kingsley, in urging the claims of the Colonial Nursing Association to the support of the community, made a mistake in describing it as "part of Mr. Chamberlain's noble scheme to stop the waste of life that goes on in the Tropics." As the Colonial Secretary would be the first to admit, the scheme, noble and admirable as it is, was that of Mrs. Francis Piggott; and it is curious that after the full account of the origin, rise, and progress of the Colonial Nursing Association, which was so lately given in these columns, Miss Kingsley should have fallen into such an error. For the rest, Miss Kingsley in no respect exaggerates the urgent need of the best trained nurses in West Africa, and we cordially echo her words that "it is a reproach to us at home that many a man's life is wrecked, and many a death caused, by the want of a little nursing." This is one of the pressing wants which the Colonial Nursing Association has set itself to meet, and its complete success is certain if only the public will render the essential financial assistance.

### NURSING OF THE SICK POOR AT SHEFFIELD.

The tenth report in connection with St. George's Home, Sheffield, for district and private nursing contains several melancholy passages. "Owing to the outbreak of typhoid the number of cases far exceeded the powers of the district nurses. There was wholly inadequate hospital accommodation, so that the cases could not be removed. . . . The patients could not be left to die unattended; the only thing to be done was to put an extra nurse when necessary. . . . This necessity has been the cause of much difficulty and heavy expense. . . . There has also been much sickness among the nurses themselves, and the year has closed in debt." In additional respects the year has been a sad one. The lady superintendent died at the commencement, and other friends have since passed away. But the record of work done is admirable. No less than 1,362 cases were nursed, and 22,362 visits were paid. There were 100 cases of typhoid. It is to be hoped that the contemplated necessity of withdrawing one of the nurses owing to insufficiency of funds will not have to be realised, and that Miss Corvan, the present energetic lady superintendent, will obtain the financial help she needs from the well-to-do Sheffield people.

### THE DANCING QUESTION AT LAMBETH.

The Lambeth Board of Guardians declined to prohibit the annual dance at the infirmary, though the matron offered her strongest opposition to the perpetuation of the gathering. She urged that her experience was that dancing tends to the disorganisation of discipline, and that it is detrimental to the working and tone of the institution. It will be seen that, according to a letter elsewhere, written "on behalf of the nurses," there is no truth in the widely-circulated statement that the nurses threatened to strike in the event of the ball being prohibited. We welcome this contradiction, partly because we greatly object to the idea of strikes, and partly because we are by no means certain that these so-called "nurses' dances" are desirable institutions.

### THE NURSES OF THE HALIFAX WORKHOUSE.

As an illustration of the interest which the nurses of the Halifax Workhouse take in their work, a local paper mentions the fact that they provided, with their own money, the neat sitting-up gowns which were worn by some of the patients at a distribution of welcome gifts by the Mayor and Mayoress, the frills adorning the bedding in certain instances, and the flowers beautifying the wards. As to the last, we suppose it is true that few people, when they have flowers to distribute, remember the Workhouse Infirmary. Yet there are few places in which they are more appreciated.

### THE NIGHT NURSING QUESTION AT STOCKTON HOSPITAL.

The attention of the General Committee of the Stockton and Thornaby Hospital has very properly been called to the question of night nursing. With an average of about 50 patients to look after there are only two night nurses, the result being, as Dr. Deneham stated at a meeting last week, that it sometimes happened that these were called to the out-patients' wards, and the hospital itself was left unprotected. He instanced a case that recently occurred. It was one which required constant nursing, and whilst the nurse left the patient for a few moments the latter got out of bed, with great risk to herself. The matron would have got a special nurse, only, unfortunately, they had no sleeping accommodation for her. This is a condition of affairs that ought not to exist in such a prosperous town as Stockton. In the course of the discussion it was observed that the matron objected some time ago to having a nurse to sleep out, and that she would probably object again. But the right course to pursue is obviously to provide the accommodation essential for a sufficient staff of night nurses.

### THE HOSPITAL AND HOME FOR INCURABLE CHILDREN.

One of the brightest and pleasantest of seasonable fêtes was provided for the little ones at the Hospital and Home for Incurable Children, Maida Vale, last week. Mrs. Bruce and her nurses had tastefully decorated the whole of the house, and arrayed the inmates in their holiday garb of red frocks and blouses. A large number of friends were present, and the children themselves opened the proceedings by singing a selection of carols. Their performance was creditable to their instructress, Miss F. Webb. One of the carols, entitled a "Cradle Song," was composed for them by the Hon. Chaplain to the Home. Miss Bacon's Ladies' Mandoline and Guitar Band performed effectively, Mrs. Odell singing "The Absent-minded Beggar." The audience was extremely sympathetic, for the war has excited the greatest interest amongst these crippled sufferers. Tea was served in another room, and many of the guests took the opportunity of inspecting the arrangements made for the comfort and instruction of the inmates. The Committee of Management are seeking to raise an Out-patient Fund, in order to be able to send the inmates, when they reach the age of 16, and are no longer eligible for the Children's Home, into suitable institutions.

### A MUSICAL EVENING AT A NURSES' HOME.

The nurses of the Whiston New Infirmary, Prescot, thoroughly enjoyed a musical evening given by the lady superintendent last week. The home was very prettily decorated by the nurses, and was very much admired by the guests, who also thoroughly enjoyed the evening. There was an excellent programme, and supper was provided during an interval. The mandoline band was an especial attraction, the children looking very pretty in their bright costumes.

### A SIMPLE APPLIANCE.

Most nurses who have the care of children experience a difficulty in keeping them safely and comfortably in their cots. Numerous grievous accidents have come to pass through a tumble out of bed, and others through ineffectual attempts to tie young children in. At the Children's Hospital, Shadwell, an excellent contrivance is in use which is as simple as it is cheap. The matron has amongst her stores a supply of scarlet webbing about two and a-half inches broad, and the nurse's nimble fingers speedily stitch it into shape. Two strips, amply large, are made into circles for the arms, and joined together by a strap across the chest. This is put on the child as a toy bridle would be if it were playing at horses; then a long strap is passed through the armholes at the back, and the ends are tied underneath the cot. There is no pressure anywhere, and the child has a certain liberty of movement, but it cannot get up and throw itself out of bed the moment the nurse's back is turned. The home sister at Shadwell is kind enough to say that she would gladly show the contrivance to any nurse interested, and give her a pattern if requested.

### SHORT ITEMS.

It is stated that several ladies in Calcutta have offered to proceed to the Cape on nursing duty, but their qualifications for the work are not indicated.—The total amount collected by Nurse Roberson, of Durban, for the Junius S. Morgan Fund is £6 15s. 6d., not £5 15s. 6d.—At the annual meeting of the Darlington Queen's Nurses' Association it was announced that the debt of £609 upon the Nurses' Home had been cleared in connection with subscriptions for a memorial to the late Mr. Arthur Pease.—The second sessional lecture under the auspices of the Royal British Nurses' Association will be delivered at 11, Chandos Street, Cavendish Square, on Thursday evening, January 25th, at six p.m., by Mr. Sidney Spokes. The subject will be "Impressions of the Transvaal," and the lecture will be the league. — A leather screen, representing the "Maine," has been presented to the American Ladies' Hospital Ship Fund by Mr. J. Wilfred Morris, of 17, Berners Street, and will be on view until the 9th of next month, free of charge. The screen, which was designed and executed by Mr. Morris, represents the "Maine" leaving England, and is to be raffled for, the proceeds being handed to the Hospital Ship Fund.

### Lectures to Nurses.

By CARSTAIRS DOUGLAS, M.D., B.Sc.Edin., Professor of Medical Jurisprudence, Anderson's College Medical School, and Dispensary Physician, Samaritan Hospital, Glasgow.

### DIETETICS AND THE PHYSIOLOGY OF DIGESTION —(*continued*).

THE digestive juice supplied by the stomach contains two ferments, named respectively pepsin (which acts on proteid foods, such as meat) and rennin, or the milk-curdling ferment; in addition to these there is always a small amount, about two parts per thousand, of free hydrochloric acid present, and various salts, chiefly chlorides and phosphates. In all parts of the stomach glands are found capable of secreting the ferments, while it is only those in the cardiac half of the organ that supply us with free acid. Towards the pylorus, or outlet from the stomach, the muscle fibres in the wall are collected into a ring which acts as a sphincter, relaxing automatically at suitable intervals to let the stomach contents escape into the small intestine. Whenever the food enters the organ the walls are thrown into activity and the food particles are moved about, not only from one end of the stomach to the other and back again, but also from the periphery towards the centre by a sort of churning motion. In this way all the food is worked into a uniform mixture and thoroughly incorporated with the gastric juice. The food is termed chyme when it has reached this stage. The special change which the food undergoes in the stomach is the conversion of albuminous or proteid foods into substances named albumoses and peptones, in which form they are suited for absorption into the system. Very soon after the food enters the stomach the pyloric sphincter begins to relax at intervals, allowing at first the more fluid portions of the food to pass out into the bowel. In this way the mass of chyme grows rather more semi-solid, until, as digestion proceeds, portions of this also are allowed to escape. In due course the stomach is completely emptied, but this does not take place, in the case of an ordinary mixed meal, till from three to five hours have elapsed.

We must now proceed to consider the anatomy of the small intestine. This, the longest part of the alimentary tract, measures about twenty feet in length, and is divided into three portions: (1) The duodenum, the part next the stomach, so called because its length was supposed to be twelve fingers' breadth (Lat. *duodecim*, twelve); its actual length is about ten inches, and it is itself sub-divided into three sub divisions —an ascending, a descending, and a transverse part. It is in the descending part that the bile-duct, and duct from the pancreas, open into the intestine.

The next part of the small bowel is termed the jejunum, and constitutes about two-fifths of the remainder of the small intestine. It receives its name from the fact that it is often found empty after death (Lat. *jejunus*, empty). The remaining and longest part of the small intestine is termed the ileum, because it is thrown into many coils (Gr. *eilein*, to coil). The bowel, like the stomach, has four coats—peritoneal, muscular, submucous, and mucous. The last-named is provided with many glands, some secreting mucin; others, such as Lieberkühn's follicles, yielding a digestive juice of feeble power; while, in addition, it is richly furnished with many delicate, minute, finger-like processes called villi, which are all-important in the absorption of food. In describing the small intestine in its relation to digestion we must also consider two very important glands which are intimately associated with it, viz., the liver and the pancreas. The liver, which occupies a large portion of the arch of the diaphragm, is the largest gland in the body (50—60 oz.), and, in addition to playing other parts of importance in the processes of metabolism, furnishes us with the fluid so well known under the name of bile. This is poured from the liver by the common bile-duct soon after food enters the stomach, and the secretion appears to attain its maximum five to eight hours after a meal has been taken It is a reddish-brown or greenish fluid, with a faint unpleasant odour and bitter taste, and is usually feebly alkaline in reaction. It contains two acids, taurocholic and glycocholic, combined with soda; two pigments, bilirubin and biliverdin; mucin, and certain other ingredients, such as cholesterin, urea, and salts. Bile itself is of feeble digestive power, but it seems to be of use in the absorption of fat, and is probably also of aid in pancreatic digestion.

This leads us to the consideration of the pancreas, which is a very potent organ in digestion, much more so, indeed, than we are accustomed to give it credit for. Its juice is far more powerful than either the saliva or the gastric secretion, and, as it contains several digestive ferments, is capable of producing not one, but several effects upon the food mass. The pancreas, or sweetbread, is a long-shaped gland lying across the abdomen, about the level of the second lumbar vertebra. Its larger end, termed the head, lies in the loop of the duodenum, and the duct which conveys its secretion opens into that part of the bowel. The tapering extremity, or tail, ends near the spleen. In structure the pancreas is very like a salivary gland, notably the parotid, and on this account it has been named by the Germans the Bauchspeichel-drüse, or belly-spittle-gland. The weight of this organ is from two to six ounces, and it measures six or seven inches in length. As we have already mentioned, the secretion of the pancreas plays a most important part in the preparation of food-stuffs for absorption. As a matter of fact, after the mass of chyme from the stomach has been subjected to its influence it may be stated that for all practical purposes digestion is completed.

No fewer than four ferments are present in the pancreatic secretion; they are as follows: (1) Trypsin, which acts on proteid food, as pepsin does in the stomach, producing albumoses and peptones, with these differences, that it acts in an alkaline not an acid fluid, and that its action is more powerful and rapid than that of pepsin. (2) Amylopsin, a ferment which, like ptyalin in the saliva, converts starch into maltose; it also is very energetic in its action. (3) Steapsin, which acts on fat in a two-fold manner, partly emulsifying it, and partly splitting it up. (4) A milk-curdling ferment, which can affect milk in the same way as rennet does in the stomach. It must always be borne in mind that the reaction of this fluid is alkaline, owing chiefly to the presence of carbonate and phosphate of soda.

The digestive fluid of the small intestine has been already referred to; it is called the succus entericus, and contains a ferment named invertin, capable of changing starch into maltose and glucose, and cane sugar into glucose. It has practically no effect on proteids and fats. Bacteria flourish excellently in the small bowel, producing various decomposition products from the food, such as fatty acids, leucin, tyrosin, carbonic acid, and hydrogen gas. Although the intestinal and pancreatic juices are alkaline, the formation of acids by bacteria makes the contents of the bowel acid in reaction. The ileum joins the large intestine at the cæcum, from which arises the colon, divided into ascending, transverse, and descending portions, the latter of which terminates in the rectum. The mucous lining of the large bowel is much smoother than that of the small; practically little digestion takes place here, but absorption of peptones, sugar, and water continues, and bacterial action is vigorous. The food, or rather the residue of it, which is to be thrown off from the body as the fæces, here begins to have a disagreeable odour, owing to the formation of such substances as indol, skatol, and phenol.

### Nursing the Wounded at Woolwich.

### A VISIT TO HERBERT HOSPITAL.

(By a Special Correspondent.)

Through the courtesy of Colonel Bourke, R.A.M.C., the Registrar and Secretary, I had the opportunity last Friday of going over the Military Hospital at Woolwich, of seeing the wounded from South Africa, and of talking to one of the Army Sisters.

It was a lovely bright day, and as I approached the end of the three-mile drive from Blackheath Station—the hospital is in reality situated on Shooter's Hill, and is therefore nearer to Blackheath than to Woolwich—I was much struck with the situation of the home where "Tommy" is sent when he is sick. All about are green fields and open spaces, the air blows fresh and sweet, and the quiet must be of distinct benefit to those who have returned ill and overwrought from the din and bustle of life at the front, or who have been overtaken by disease whilst in London barracks. The solid-looking pile of grey stone looks venerable and impressive, more especially in contrast to the new red blocks of the Brook Hospital, which is not very far away.

### The Hospital Not Full.

As we crossed the quadrangle leading from the entrance gates to the building itself, which in spring will be gay with tulips and crocuses, and in summer ablaze with thousands of scarlet geraniums, I asked my cicerone whether the hospital was very full at present.

"No," was the reply, "we can accommodate about 700, whereas our present numbers are only about 400, and of these at least a hundred could be discharged almost at once, their health is so nearly re-established."

"You do not then get rid of the men as soon as they are well enough to leave, Colonel Bourke?"

"My policy has always been when practicable to keep them a little time after they are apparently well. It sometimes happens that unsuspected complications arise later, which would necessitate a return to hospital quarters, and anyhow a man frequently wants a good deal of extra care and extra nourishment even after he has been pronounced cured. This extra attention he could not obtain either in his own home or back at his duty, and accordingly a good many of our patients are really convalescents."

"And the men from South Africa, are they progressing favourably?"

"Admirably; but you shall see for yourself later on."

### The Enteric Cases.

"Amongst the most serious, I suppose, are the enteric cases, of which it is stated you have a great number?"

"That is a mistake. We have only two enteric cases in the place. One is convalescent and has returned to ordinary diet, the other's temperature to-day is normal. Neither of these men has been to South Africa. Those who were sent here from the seat of the war were men long since convalescent from the disease, which they contracted last October. Many have been sent back to their duty, and the remainder will go in a day or so."

### The Nursing Staff.

Here we were met by Sister Tulloch, who kindly offered to accompany us round the wards, and I availed myself of the chance of ascertaining the strength of the nursing staff.

"Seven sisters, a superintendent sister taking the place of a matron. We are assisted by 130 non-commissioned officers and men, who act as orderlies."

"I suppose that nearly all the staff are new, as so many were called for active service?"

"I myself have been here three years," said Sister Tulloch, "but six army sisters went out to South Africa, one of whom is now inside Ladysmith, in the hospital."

"And you also lost nearly all your experienced orderlies, I believe?"

"Not at all," replied Colonel Bourke. "Fifty of the Royal Army Medical Corps are still here. These are supplemented by regimental orderlies, who are specially selected. Nearly all have been previously trained in hospital work. They are assisted by the Militia medical staff, who have a month's training every year in this very hospital."

### The Patients' Wards.

By this time we had passed into one of the wards, still decorated for Christmas. The afternoon light was streaming in pleasantly through the many windows. A few of the beds were occupied, but many of the men were sitting round one of the glowing fires, chatting to each other, or lounging and resting whilst perusing some of the numerous magazines taken from a table not far distant. The air seemed very pure in spite of the fact that the rooms were cosily warm to counteract the outside frost. The stoves, of which there are two in each ward, give out a splendid heat. In addition to these, the hot-water pipes were working. At the end of the wards, on the outside wall, are the lavatories; at the other end is the sisters' room. Thus, as there are two wards in each pavilion, the sisters' rooms are sufficiently near for the occupants to be of use to each other if need should arise, both being in the centre of the block.

### The Hours of Duty.

I asked Sister Tulloch the hours for the sisters to be in the wards.

"Always," she replied. "That is, there is always a sister on duty, night and day. In serious cases, especially of enteric or pneumonia, during the most anxious time the sufferers are never left by the sisters, one relieving the other till the crisis has passed. A special orderly is told off to assist the sister; but washing, feeding, and all duties are performed by the sister should occasion require."

"But, is it not a rule that you should not remain in the wards?"

"On the contrary," said Colonel Bourke, "the rules of the hospital oblige, rather than forbid, the sisters to remain in the wards. They are free to do anything which will conduce to the comfort and well-being of the patients. The object of the hospital is to cure the men committed to its care, so that they may, if possible, be able to return to their posts."

### The Wounded Soldiers.

As we went into another ward 43 beds were pointed out to me all ready for the men from the front who are due to arrive on the 23rd of this month, and then we came to an interesting group of those who had returned a few weeks back. Some were from Estcourt, others from Elandslaagte or Mooi River, or Colenso, but all were up, and all seemed on the high road to recovery. Upon asking one man where he had been wounded, he rolled his trousers to his knee, and showed me close to the bone on the front of the leg the spot where the bullet had entered. Taking an upward course, it had made its exit at the back, a little higher than the knee. I never saw such a neat wound. Neither of the holes had left a scar larger than that of a very small pea, and there was not the slightest roughness or ridge on the skin, only a slight dark discoloration. The leg and knee were as pliable as before injury. I observed the same perfect healing in another man who had been shot through the hand, and in one whose foot had been pierced. The latter showed me the boot that he had been wearing at the time, which he kept handy near his bed. The bullet had gone in at the front of the foot, making a scarcely visible hole in the leather, but it had torn the vamp slightly at the back in working its way out. One poor fellow was still much bandaged, having lost two fingers by the explosion of his gun; but a bad bayonet cut in the head of another man, caused by a fall when charging over uneven ground, had healed so well as to be hardly perceptible. The men were too modest to make much of their experiences, but the sister told me that they sometimes held forth to a delighted audience of their fellow patients and sisters. Several were hoping to get back before long.

The Diet for the Patients.

After a glimpse of the library, where the men were reading and writing letters, I was conducted into the kitchens. The floors were spotless, the stoves shining, and everything was delightfully neat. I arrived at an opportune moment, for fifty or sixty custards, in small oblong tin dishes, had just been turned out of the oven. Each one was browned to a nicety, each was firm and refused to move by shaking, the smell being most appetising. As we left Colonel Bourke told me they had all been made with eggs, egg powder was never used in the hospital, it not being considered sufficiently nutritious. I inquired as to meals. Breakfast is served at nine, dinner at one, and tea at five, with milk and beef tea at intervals as ordered by the doctor. The latter is warmed at night by the sister in her room, and taken by her into the wards. As further evidence of the way "Tommy" is fed, I may mention that the chicken bill for the invalids is £150 a month; and when it was thought that the head cook was not quite all he might be in the matter of preparing tempting viands he was sent for four months to the Constitutional Club in London to get the necessary experience.

### The Nurses of the Royal Free Hospital.

### A CHAT WITH THE MATRON.

By Our Commissioner.

The nurses' quarters at the Royal Free Hospital are quite separate from, though communicating with, the main building. The block in which they are situated, however, is not entirely devoted to one purpose. The nurses have the basement for their use, but not the ground floor, and the office of the Matron is on the first floor. Here, the other day, I found Miss Wedgwood, who had courteously consented to see me for a chat about her staff. As I knew that the question of additional accommodation for the nurses was under consideration by the committee, I referred to the subject at the outset.

"We have not a sufficient number of nurses to work the hospital in accordance with the present standard of nursing," said the Matron, "but the accommodation we possess does not admit of any further increase in the staff. We are really in a transition state at present. A scheme for rebuilding this department has been under discussion for the past two years. The question is whether we shall do away with all the cubicles."

"What is your opinion about it?"

"I wish the scheme to embrace a separate room for each nurse. In that event the job will be done once for all. Our only difficulty is the want of the necessary funds. If we could get the money we require, we should soon have the nurses' quarters enlarged and improved."

"How large is your existing staff?"

"There are forty-nine nurses, and we want rooms for fifty-six. I should very much urge an immediate increase if we only had the requisite room. However, we should get on very well with the staff we have if it were not for the nursing of cases in the isolation wards and special cases. Allowance is made for illness among the nurses."

"I should like to know the percentage of patients to each nurse?"

"There is one nurse to three and a-quarter patients—not a very big allowance. It would be better to have one to three or two and three-quarters. I think that at some London hospitals the proportion is one to two and three-quarters.

"What is the average number of patients?"

"Until 1899 it has been 140 or 142, but several wards are at present closed for rebuilding purposes. During the past twelve months, new kitchens, lavatories, and linen-rooms have been added to the three wards of the Victoria wing, and on that side of the hospital the accommodation has been very much improved."

"When were your rules for probationers and regulations last altered?"

"They were revised in April, 1894. As you are aware, the training here is for four years."

"Do you find the system work well?"

"Yes. Of course, a good training is obtained in three years, but in the fourth year the nurses are able to take the work of a night or day Sister, or casualty work. We always keep one of the nurses in her fourth year on duty in the casualty receiving-room."

"According to your experience, do the nurses themselves like the four years' training?"

"I think that they do. We have never had any difficulty about it. I only remember one family asking me, tentatively, if the nurse could be released at the end of three years."

"You do not release them before the close of the four years?"

"Only in the event of illness or under special circumstances. This is not a very suitable hospital for delicate people; there is too much exposure crossing the quadrangle, and the nurses have to work very hard. We have had two cases of delicacy of the lungs, and in these the committee released the nurse and presented a certificate at the end of three years."

"It is sometimes urged that there is a tendency to make the commercial element too prominent in nursing. Do you consider that there is any foundation for it?"

"I do. Nursing was never intended to be merely commercial, and I deprecate any tendency in that direction. The principle of 'so much work so much money' is very good up to a certain point, but the work is not such that it can be done for money only without lowering it a great deal. Indeed, I maintain that it is utterly ruined if it is looked at from a purely commercial standpoint. Probably this sort of thing is more noticeable in private nursing than in hospitals."

"Do many of your nurses stay after their four years' training?"

"Those for whom we have employment as Sisters are anxious to remain. It is a great advantage for a nurse to have been a sister in her own hospital. We do not keep the nurses after their training unless we have vacancies for sisters. The four years' system prevents us from having vacancies for staff nurses. Probationers are admitted between the age of 23 and 35 on three months' trial."

"How many beds are there in a ward?"

"The usual number is eighteen, and these are nursed by a sister, a senior and a junior probationer. Very often the senior is entitled to the designation of nurse. The male accident ward has only sixteen beds, but the operating theatre is attached to it. On the other hand, one ward has an isolation ward adjoining, with two beds, making twenty."

"What extra leave do the nurses have?"

"The nurses on day duty get one day, from seven a.m. to ten p.m., in each month, but the nurses on night duty, I am very sorry to say, only get two days in three months. But we could not, with our present staff, and with the drain from the isolation and acute cases, give the night nurses a day a month. As to holidays, probationers in their first year have sixteen days; nurses and probationers in their second, third, and fourth years three weeks; and sisters four weeks per annum."

"I believe you have a pension fund for nurses?"

"Yes, the committee assist all nurses who desire to join the Royal National Pension Fund by paying one-half of their annual premium whilst they remain in the service of the hospital. These premiums are paid into the returnable fund of the Royal National Pension Fund to secure a pension of £22 10s. per annum, to commence at the age of 50, 55, or 60 years."

"Your female medical students have lately been very successful?"

"They have done well at the recent London University Examinations for the M.B. degree. The number of students varies, but the entries just now are very large."

"Is the health of the nurses good, generally speaking?"

"We have enjoyed great immunity from serious illness. Our rules are so stringent that it is very difficult for anyone to contract disease. In eight years there has not been a single death of a nurse in the hospital. We had a small outbreak of typhoid fever in 1897, the sufferers being one nurse, three medical officers, and the housekeeper. They were in different parts of the hospital."

"Did you ever ascertain the cause of the outbreak in 1897?"

"No. Inquiries were made by the medical staff and by the lay Board as to the milk supply, and the Medical Officer for St. Pancras carried out a searching investigation, but to no purpose."

"Do you consider that the nursing of typhoid cases is attended by much risk?"

"No nurse ought to get typhoid. Our regulations are so definite and so stringent that, if they are strictly adhered to, there is substantially no danger."

"Is any form of out-door recreation provided for them?"

"Unfortunately, no. I do not encourage cycling because Gray's Inn Road is so unsafe for it; there is no room for a tennis court. The Benchers kindly allow them to use Gray's Inn Gardens. I am most anxious when we obtain our new building to have flat roofs for out-door sitting-rooms."

Talking for a few minutes with Mr. Conrad Thies, the secretary, I learnt that there is a reasonable prospect of something being done to improve the nurses' quarters shortly. The Prince of Wales's Fund has just made a special grant of £500 towards the cost of the work, which is, however, estimated to cost at least £5,000. It is a feature at the Royal Free Hospital for members of the Ladies' Visiting Committee, to make a monthly report to the weekly board. These ladies dine occasionally with the nurses. Glancing over the comments of the visitors, which are recorded in writing, I observed that they were of a very favourable character, and such as to justify the conviction that the nurses have little to complain of in the matter of diet.

### Appointments.

HANWELL COTTAGE HOSPITAL.—Miss Adela d'Arcy has been appointed Nurse-Matron. She was trained at Sussex County Hospital, Brighton, and has since been nurse at the Suffolk Hospital, Bury St. Edmunds, the General Infirmary, Worcester, the Royal Infirmary, Aberdeen, the Staffordshire Hospital, Wolverhampton, and Andover Cottage Hospital. She has also been district nurse at Gloucester, and nurse-matron at the London Throat Hospital and the Malmesbury and District Cottage Hospital.

### Minor Appointments.

ROYAL BERKS HOSPITAL, READING.—Miss Martha Lamble has been appointed Sister. She was trained at the North Staffordshire Infirmary, where she was subsequently charge nurse. She has also been sister at Ulster Hospital for Children and Women, and sister at the Guest Hospital, Dudley.

CITY OF WORCESTER ISOLATION HOSPITAL.—Miss Frances Cann has been appointed Charge Nurse. She was trained for four years at the Royal Nursing Institution, Derby, and has since been charge nurse at Stockport Isolation Hospital and Tonbridge Union Infirmary.

ROYAL VICTORIA HOSPITAL, BOURNEMOUTH. — Miss Marianne L. Floyd has been appointed Charge Nurse. She was trained for four years at Croydon General Hospital. She has since been sister of the Durham County Hospital, and sister of the new corridor of eighty beds of medical and surgical cases, with the care of the operating theatre.

BRIGHTON WORKHOUSE INFIRMARY.—Miss Agnes R. McInnes has been appointed Charge Nurse for sick children. She was trained at the Walton Hospital, Liverpool and has since been nurse at the West Derby Union Workhouse, Walton.

CUMBERLAND INFIRMARY.—Miss Martha Edwards Pratt has been appointed Staff Nurse. She was trained for three years at Leeds Union Infirmary, and has since been matron's assistant in the same institution.

VICTORIA HOSPITAL, KINGSTON-ON-THAMES.—Miss Jean Frances Vincent has been appointed Charge Nurse. She was trained at Bolingbroke Hospital, Wandsworth Common, and has been night charge nurse at Muswell Hill Fever Hospital.

LINCOLN UNION WORKHOUSE.—Miss Catherine Clarke has been appointed Superintendent Nurse. She was trained at Paisley Infirmary.

### Presentations.

POPLAR AND STEPNEY SICK ASYLUM.—Miss Joan Inglis, senior assistant matron at the Poplar and Stepney Sick Asylum, has been presented with a handsome Russian leather dressing-bag (silver-mounted fittings), a leather case containing silver button-hooks and shoe-lift, and a set of silver filigree tea-spoons and sugar-tongs from the officers and nursing staff; also with a dainty Minton china afternoon tea service from the domestic staff. The presentations were made in consequence of her retirement to fill the post of matron at the Leeds Union Infirmary, and in token of the high esteem and gratitude felt by all who have worked with her. Her kindness and untiring energy endeared her to the whole staff, and she has their heartiest wishes for her success and happiness in her new sphere.

HOUGHALL INFECTIOUS DISEASES HOSPITAL.—On Saturday afternoon Nurse Essex, who for between six and seven years has worked with great diligence and success under the Durham Rural District Council at the Houghall Hospital, was presented by the chairman of the council with a handsome travelling clock and a gold brooch. Both the Chairman and Dr. Jepson testified, in the most cordial manner, to the patience and kindness manifested by Miss Essex towards the patients, the latter observing that "she had made the hospital quite a little heaven for children." Judging by all the nice things that were said about her on the occasion, it is a matter for congratulation that she has obtained an appointment at Guy's Hospital.

IPSWICH NEW INFIRMARY.—On Saturday Miss Bessie Kemp, charge nurse of the Ipswich New Infirmary, was presented with a silver cruet, set of glass dishes, silver butter-knife, tablecloth, and silk cushions from her colleagues, as a mark of the esteem in which she is held. The occasion is her retirement owing to her marriage.

### The Princess of Wales's War Fund.

We publish to-day a further list of subscriptions from nurses to the Princess of Wales's War Fund, and remind our readers that the subscription is limited to five shillings. All subscriptions should be addressed to the Manager, Office of The Hospital, 28 and 29, Southampton Street, Strand, London, W.C., and the name and address should always be given. The list will close at the end of the month.

Amount already acknowledged, £193 6s. 3d.

| | s. | d. |
|---|---|---|
| Policy 2,591, P.F. | 5 | 0 |
| F. Staunton, P.F. | 5 | 0 |
| P. Crispin, P.F. | 5 | 0 |
| E. Potter | 4 | 0 |
| Policy 352, P.F. | 3 | 0 |
| Policy 6,386, P.F. | 3 | 0 |
| Frances M. Hart, P.F. | 2 | 6 |
| S. L. Cooke, P.F. | 2 | 6 |
| T. A. Leverett, P.F. | 2 | 6 |
| D. C. Thomas, P.F. | 2 | 6 |
| M. J. Millard, P.F. | 2 | 0 |
| Nurse Gibbard, P.F. | 2 | 0 |
| Jennie Jones, P.F. | 2 | 0 |
| A. Poston, P.F. | 2 | 0 |
| Matilda McLatchie, P.F. | 2 | 0 |
| E. Cooper | 1 | 0 |
| Mrs. Tryphena Tope, P.F. | 1 | 0 |
| M.L. Collingwood, P.F. | 5 | 0 |
| Mary S. Crossland, P.F. | 5 | 0 |
| S. A. Bell, P.F. | 5 | 0 |
| Maria Newman, P.F. | 5 | 0 |
| Nurse Lucy | 5 | 0 |
| Miss Shaw, P.F. | 5 | 0 |
| E. W. Mowat, P.F. | 5 | 0 |
| M. Carr, P.F. | 5 | 0 |
| Katherine Reeve, P.F. | 3 | 6 |
| H. Preston, P.F. | 2 | 6 |
| Policy 3,563, P.F. | 2 | 6 |
| Florence Standley, P.F. | 2 | 6 |
| E. Davies, P.F. | 2 | 6 |
| J. E. Parsons, P.F. | 2 | 0 |
| F.C., Policy 6,705, P.F. | 2 | 0 |
| A. Burgess, P.F. | 1 | 6 |
| Policy 6,176, P.F. | 1 | 0 |
| Policy 4,997, P.F. | 2 | 0 |
| A. M. Howells, P.F. | 5 | 0 |
| E. Carter, P.F. | 1 | 0 |
| A. M. Burke, P.F. | 5 | 0 |
| M. Whitehead | 5 | 0 |
| Nurse Sayers, P.F. | 5 | 0 |
| Sister King, P.F. | 5 | 0 |
| L. Shepherd | 5 | 0 |
| Alice | 5 | 0 |
| E. Purvis, P.F. | 5 | 0 |
| F. W. Taylor | 5 | 0 |
| E. A. Wilkinson | 5 | 0 |
| Miss Sara Wilson, P.F. | 4 | 6 |
| Clara Eckles, P.F. | 4 | 0 |
| E. L. P. Chetwund | 3 | 6 |
| Nurse Hammond, P.F. | 3 | 0 |
| A. Cameron, P.F. | 3 | 0 |
| A. O. Dickinson, P.F. | 2 | 6 |
| K. Cox, P.F. | 2 | 6 |
| Mary A. Muzeen, P.F. | 2 | 6 |
| A. E. Homan, P.F. | 2 | 0 |
| Margaret Dalziel, P.F. | 2 | 0 |
| A. B. Ash, P.F. | 2 | 0 |
| C E. Whistler, P.F. | 2 | 0 |
| Rebecca Browning | 2 | 0 |
| Nurse Orton | 2 | 0 |
| Nurse Macfarlane | 2 | 0 |
| Nurse Priss, P.F. | 1 | 6 |
| F. E. B., Policy 3,532, P.F. | 1 | 0 |
| R. W. G., Policy 5,362, P.F. | 1 | 0 |
| Nurse Parsons | 1 | 0 |
| Policy 5,714, P.F. | 1 | 0 |
| Patients and Nurses of a Hospital Ward | 6 | 6 |
| Emma J. Haddock, P.F. | 5 | 0 |
| M. L, Policy 2,901, P.F. | 5 | 0 |
| A. M. Waterfield, P.F. | 5 | 0 |
| Alice Arnold, P.F. | 5 | 0 |
| Policy 1,816, P.F. | 5 | 0 |
| F. M. Gregson, P F. | 5 | 0 |
| James Grant, P.F. | 3 | 6 |
| Sister Vernall, P.F. | 3 | 0 |
| C. Macdiarmid, P.F. | 3 | 0 |
| S. A. Bickford | 2 | 6 |
| J. M. Field | 3 | 0 |
| A. Fairlie | 1 | 6 |
| Sister Victoria | 1 | 1 |
| Mary C. Beavan, P.F. | 1 | 0 |
| Policy 1,479, P.F. | 1 | 0 |
| A. L. Dobson | 1 | 0 |
| E. A. Wilson, P.F. | 5 | 0 |
| Nurse Jacobs | 5 | 0 |
| Bessie Keillor | 5 | 0 |
| Policy 62, P.F. | 2 | 6 |
| E. | 2 | 6 |
| Nurse Parsons | 2 | 6 |
| H. M. Turner, P.F. | 2 | 6 |
| Policy 4,352, P.F. | 2 | 0 |
| A. M. Scholes, P.F. | 2 | 0 |
| Nurse Constantine, P.F. | 2 | 0 |
| M. J. Harris, P.F. | 1 | 0 |
| Policy 2,615, P.F. | 1 | 0 |
| Nurse Burley | 1 | 0 |
| R. H. N. Giles | 1 | 0 |
| M. Coverdale, P.F. | 1 | 0 |

Collected at the Birmingham City Asylum.

| | s. | d. |
|---|---|---|
| J. K. Dinwoodie, P.F. | 5 | 0 |
| G. Winterbottom | 1 | 0 |
| Miss Taylor | 4 | 0 |
| E. French | 1 | 0 |
| E. Betterley | 1 | 0 |
| Mrs. Watkinson | 1 | 0 |
| Millicent Cash | 1 | 0 |
| Mary Nicholas | 2 | 0 |
| Nurse Buncle | 1 | 0 |
| Nurse Pearson | 1 | 0 |
| Nurse Cobley | 1 | 0 |
| Nurse Williams | 1 | 0 |
| Nurse Duffield | 1 | 0 |
| Nurse Hodgetts | 1 | 0 |
| Nurse Basterfield | 1 | 0 |
| Nurse Lewis | 1 | 0 |
| Nurse Wright | 1 | 0 |
| Nurse Baker | 1 | 0 |
| Nurse Thompson | 1 | 0 |
| Collected by Nurse Buncle | 5 | 0 |

### The Absent-Minded Giver.

When you've answered Rudyard Kipling—when to Atkins' wants you've seen—
When you've finished sending Tommy pipes and socks
Will you kindly save a shilling from that "little tambourine"
To drop into another little box?
You're an absent-minded giver, but your kindnesses are great,
And you give to all good objects when you find them.
And, though Tommy's out on service, the sick and poor still wait
For the little lot of "sovs" it costs to mind them.
Sick Box, Poor Box, Box of a Gallant Boat,
(Fifty thousand willing hands working from day to day!)
Each of 'em doing its noble work (and who's to keep 'em afloat?)—
Pass the hat for your credit's sake, and pay—pay—pay!

There are homes for convalescents and orphanages, too,
That need a hand to help them in their stress;
There is gas, and coals, and "vittles," and the wages falling due,
And it's more than rather likely there's distress.
There are folks you gave to casual, they'll be sorry now you've missed,
For an absent-minded giver they will find you;
Now that War Funds are the fashion, you have left them off your list,
And to answer their appeals you have no mind to.
Girls' Homes—Boys' Homes—Homes for the Deaf and Dumb—
Homes for Consumptive Invalids—it's all the same to-day!
Each of 'em doing its noble work (and who's to give 'em a crumb?)—
Pass the hat for your credit's sake, and pay—pay—pay!

There are hospitals by hundreds far too poor to make a spread,
And they'll put their sticks and bedding up the spout,
And they'll live on half o' nothing till their inmates are half dead,
'Cause the source that sent the "subs" has given out.
You're an absent-minded giver, but you heard your Kipling call,
And your Lord Mayors didn't need to send to find you;
You dropped your "subs" and joined them, so the job before us all
Is to help the funds that you have left behind you.
Boys' Homes—Girls' Homes—Homes for the Halt and Blind—
Homes for Decrepit Veterans—it's all the same to-day,
Each of 'em doing its noble work (and who's to keep 'em in mind?)—
Pass the hat for your credit's sake, and pay—pay—pay!

Try and manage so as later you can look them in the face,
And tell them—what they very much prefer—
That while you helped the soldiers you also kept their place,
And your contribution list did not defer.
You're an absent-minded giver, and you may forget it all,
But we do not want the needy to remind you
That you sent 'em to the workhouse 'cause the Rudyard Kipling call
Abstracted all the cash you had behind you.
Poor Box—Sick Box—Box of the Children's Friends—
(Fifty thousand willing hands working from day to day),
Each of 'em doing its noble work (and who's to make 'em amends?)—
Pass the hat for your credit's sake, and pay—pay—pay!

—*To-Day.*

### Echoes from the Outside World.

### AN OPEN LETTER TO A HOSPITAL NURSE.

A LETTER from Natal which is just to hand has a prophetic ring about it. It was written on December 18th, and the information came direct from the Boer ranks. The burgher, whose epistle was sent from the scene of action to Durban, was evidently much impressed by the preparations being made by the English for celebrating Christmas, and the following words throw a curious light on future events: "We expect," he says, "that after having eaten his fill the British soldier will be quite unfit to fight, and I think we are likely to take advantage of this fact and smash Ladysmith up." The Boers reckoned without their host this time, did they not? If our fellows had a good square meal for once on December 25th, how glad they must have been, for they certainly have a rough time of it. The last letter from the front, written just after the disaster at Tugela (pronounced Too-*gair*-lah) shows that the men had never been out of their clothes and boots for a week, and that one pint of water was all that was allowed per day for washing purposes. When one remembers that this is in the most terrific heat, and that the men sleep on the dusty, burning veldt, it is not difficult to realise that they fall an easy prey to sickness. Matters are made worse because the troops become so thirsty that they drink from stagnant pools and filthy water, with frequently, of course, enteric fever to follow. The "Lismore Castle" hospital ship, which has been fitted up at Durban, badly needed some of the gifts that were lavished on the "Princess of Wales" and the "Maine" here. The vessel had to be hurriedly prepared for the newly wounded, so that the ladies of the Durban Patriotic League were working all Sunday, making covers for splints and pads for broken limbs. The authorities appealed for shirts and pyjamas, and fortunately 250 were forthcoming at a moment's notice, owing to the industry of the League, or the poor fellows would have fared badly.

---

ENTHUSIASM and patriotism are very excellent things in their way, but it is quite possible to have too much of them, and this must have been the feeling of many of the City of London Imperial Volunteers on Saturday. Although the police authorities have had a good deal of experience as to how madly sober English men and women can behave when they have the war fever upon them, they were evidently unprepared for the enormous crowds who assembled to bid the men "God-speed." Consequently, the constables were so lamentably in the minority that they were practically at the mercy of the onlookers, who pressed forward to shake the hands of the volunteers, and to pat them on the back so often and so persistently that it is not amazing some of the men arrived at Nine Elms Station with a portion of their accoutrements torn away from them. Consequently, I presume, it is announced that there will be no passage of the next contingent through the City next Saturday, which will be a great disappointment to a large section of the public.

---

IF everyone were as averse to the employment of 'khaki' as a fashionable material as I am, I am afraid that the manufacturers who are busy devising new methods of using the soldier's cloth would lose heavily over the enterprise. It is a matter for congratulation on this particular subject, as well as on every other, that we do not all think alike, but to my mind there is something most incongruous in surrounding one's self with khaki photo frames and knick knacks, using khaki slippers and purses, and presenting one's mankind wiih khaki tobacco pouches and cigar cases. The stuff has nothing intrinsically pretty about it, and we do not surely need to be reminded in this way of our brave men who are laying down their lives for their land, attired for purposes of safety, not caprice, in the very material which we purchase with glee in any form, because it is the "craze of the moment." But the fashion is not going to be limited to trifles. Throughout the coming spring and summer children are to wear coats and overalls of khaki, the men will adopt overcoats, and the ladies will don Imperial Yeomanry hats, and coats and skirts of the fawn-coloured cloth, unless it can be demonstrated effectively to them that the dust-coloured hue is by no means becoming to any but fresh young complexions. The latter consideration would carry much weight, even with the most up-to-date fashionable dame.

---

THE exhibition of the Society of Portrait Painters is especially interesting this year because the visitor goes in the hope that she may thus gain valuable information as to the outward characteristics of some of the men who are being made notable by the events taking place in South Africa. The hope is fairly fulfilled, for in the place which last year was occupied by the Sirdar stands a most characteristic portrait of General Joubert. The portrait, which has been executed by an Amsterdam lady, Miss Therèse Schwartze, has apparently not been painted very recently, but probably the very points upon which the artist lays stress have been accentuated as the man has grown older. Everything in the picture seems square, the forehead, the face, the figure, the fingers, and even the very sheath of the sword. The same artist has another workmanlike portrait elsewhere in the gallery. On the opposite wall is "Lord Roberts," painted by Mr. Cope. It is a model portrait of a model soldier. The treatment is refined, yet not lacking in breadth; the colour is excellent, and numerous were the spectators who stood and gazed long and earnestly at the General into whose hands, after God, we have given our fate. There is assuredly nothing in the representation on canvas to shake our trust, and many of us passed on cheered by the feeling that, judged by physiognomy alone, the Field Marshal had been tried, and not found wanting. Another picture which attracts a great deal of attention is "General Sir George White," by Mr. Llewellyn The tense look upon the face would almost suggest that the portrait had been painted at the seat of the war rather than quietly at home. The firm grip of the right hand is especially noticeable, Amongst the portraits which are a pleasure to the eye—and this year they are numerous—are "Muriel," a most winsome child in red, by Mr. George Henry; "The Countess of Warwick's Little Boy," by Mr. Ellis Roberts; "Mrs. Mitchel Chapman and her Dog," by Mr. Glazebrook; and Sir L. Alma-Tadema's group of portrait heads.

---

IF any of you want to take small children to a pantomime, without a trace of vulgarity, with pretty scenes, pretty dresses, and pretty music, and which, moreover, you will enjoy as much as the juveniles, go and see "The Snow Man" at the Lyceum. One little boy, about eight, Master Hersee, is perfectly delightful. He is full of fun and impudence, never forgets his part, but is always doing something appropriate whilst others are talking, and with two comrades about the same age supplies a most charming sketch of child life. The snow scene, when the men, women, and children of the Flemish town turn out in a snow storm to build up the snow man, who afterwards came to life, with such disastrous consequences, is remarkably picturesque and pleasing.

### Everybody's Opinion.

[Correspondence on all subjects is invited, but we cannot in any way be responsible for the opinions expressed by our correspondents. No communication can be entertained if the name and address of the correspondent is not given, as a guarantee of good faith but not necessarily for publication, or unless one side of the paper only is written on.]

### A CONTRADICTION.

Nurse Ellen Moloney, on behalf of the nurses of the Lambeth Infirmary, writes: A scandalous report having been circulated in several of the leading papers to the effect that the nurses of our infirmary intended having a strike if the Guardians did not grant us a ball, I desire to contradict such a statement, as it is an abominable falsehood, on the part of my fellow nurses and self, through the medium of your paper. We do so under the plea that we were quite ignorant of any talk about the ball. We also consider that such a report will be detrimental to our nursing career, which we all desire to be a long and an honourable one.

### ST. GEORGE'S INFIRMARY, FULHAM ROAD.

"Audi Alteram Partem" writes: It is with the greatest indignation I see an article in The Hospital of January 6th inst. headed "Gross Mismanagement at St. George's Infirmary." What can be the object of this correspondent in offering such information, which, to say the least of it, is greatly exaggerated, and in some instances wholly incorrect, is beyond my imagination, and strongly advise this person, whoever he or she may be, to look more deeply into the matter another time before making such grave charges public. The nurses' diet here is excellent and sufficient, also being well served. Our clothing, to my knowledge, has never been damp, but on inquiry I learn that on one occasion it came from laundry in a damp condition, but was immediately returned. Considering an outside laundry is engaged to do the whole of the infirmary laundry, owing to a new one being in course of construction, I do not think that so very terrible. With regard to the wards being in a deplorable condition, I really do not know how such an expression could be used in reference to a ward in this institution. Any nurse who has received her training here under the present matron can testify to this, I am sure. The doctor and matron has the nurses' and patients' welfare at heart, and together they work with this ultimate end in view. We all have perfect confidence in them, and have no wish whatever for an outsider to take up the cudgels in our defence.

### UNTRAINED NURSES.

"Nurse A. M. B." writes: In reply to "C. R.'s" remarks *re* "Untrained Nurses," I should like to add a few words to "Nurse Katharine's" courteous letter, which gave me much pleasure to read. I must protest against being classed as an "impostor" even should I undertake a case (in time of emergency) otherwise than maternity. Does "C. R." know that it happens occasionally that obstetric patients develop complications, such as bronchitis, pneumonia, &c., during the lying-in period, and are we to be described as "impostors" if we stay on and nurse our patients at such a time? or are we to beat a retreat and make room for one of "C. R.'s" professional standing? Personally I should do no such thing. On three occasions I have been asked to attend cases (male), the applicants having failed to obtain a sick nurse. Before going and on my introduction to the doctor in each case I stated clearly that I was a trained maternity nurse. In neither of these cases did I receive any hint from the medical man that I was an "impostor." Quite the reverse; and from my patients I received practical proofs of their appreciation of any service I may have rendered. Maternity nurses (trained) can scarcely be said to "know nothing," and on pulse and temperatures I fancy they are as keen as their "general" sisters. As to the doctor's question *re* temperature, it is about equal to that of another medical man, who asked me, "Can you pass a catheter?"—a detail of obstetric work which is quite as much a point as pulse-taking; in fact, is one of the qualifications preliminary to sitting for the L.O.S. It does not do for nurses to be too sensitive in these days, and expect that everyone is going to look upon them as perfect compendiums of nursing details, theoretical or practical. If we are competent it will not take doctors, patients, or the general public a very long while to give us all the respect and deference which may be due to our professional ability. A little wholesome criticism is good for all of us occasionally. I cannot boast the long list of nursing certificates "C. R." does, but I claim the right to be recognised as a trained nurse in the special department for which I served as probationer for six months in a large London district, and had had ten months' practical midwifery work before sitting for the L.O.S., since which I have been kept very busy with private maternity work.

### EASY SWINDLES.

Nurse M. T. Reid writes from Blackheath as follows: Last Friday week you put an advertisement in your paper for me as "companion-nurse," and the address was that of a friend with whom I was staying. My name was given, address, 53, Kempoliott Road, Streatham Common, S.W. I had many replies, and many people to see me. One lady called, gave me her name as Mrs. Maxwell, and said her husband was a doctor living at Tring, and that they were friends of the Rothschilds. She wanted me as companion to her mother to take her abroad. She came first in the morning and returned in the afternoon at four, saying "she had dropped a cheque," but we could not find it, and she borrowed £2 from me. I have heard nothing of her since then, though I have written both to Dr. and Mrs. Maxwell at Tring, and my letters have not been returned. I have a friend who wrote to the Post-Master, and he says "such people have never lived at Tring." This person (Mrs. Maxwell) told me "she saw my advertisement in your paper." I am writing to tell you this, so that, if it is in your power, you may warn others against such impostors.

Miss Ruth Wood, of the West London Association of Certificated Nurses, 90, Hammersmith Road, writes: Seeing "Nurse's" letter in The Hospital about a lady getting £1 under false pretences, I would like to tell you what happened to me about a month ago. A lady called who was evidently a gentlewoman by birth and education. She said she had suddenly come up from Bedford and wished to get her mother into a nursing home, as she had been cablegrammed for from Africa, that her husband, who stood by young Cavendish when he had his legs shot off, was ill, though not wounded. She was closing her establishment, putting her seven servants on board wages, as her mother suffered from chronic rheumatism and would not be troubled with housekeeping. I showed her my rooms, and she said they were not large enough. But she added, "Providence has sent me, as I have an aged governess whom they would suit. She has a certain income, &c." I took her back to my office, and she went to get her bag, when she suddenly remembered she had left it in the cab. She asked if she might wait to see if the cabman returned with it, and while waiting for an hour she conversed freely. The return of the cab seemed hopeless, and as it was Saturday and she had to start for Africa first thing Tuesday, when she asked me to lend her the third-class fare to Bedford, I thought of the poor officer ill in Africa and the many little things she would wish to take. So I lent her £3. As it was not returned I wrote to Scotland Yard, and a detective came and said he believed she was known to the police and was a lady by birth and a trained nurse. But they could not do anything unless I would prosecute. As I found it might cost me £50 I let it drop. The woman is short, stout, pallid, and was dressed in grey and black hat.

### Wants and Workers.

Kimberley.—If this notice meets the eye of any old Kimberley nurse of 1892, 1893, 1894, and 1895, and she would care to go out again to the Cape, will she write to "Paddy" (using her old name), 17, Rue de Castellane, Paris?

### For Reading to the Sick.

"Whatsoever is right, that shall ye receive."—*St. Matt.* xx. 4.

When all the overwork of life
Is finished once, and fallen asleep,
We shrink no more beneath the knife,
But having sown prepare to reap;
Delivered from the crossway rough,
Delivered from the thorny scourge,
Delivered from the tossing surge,
Then shall we find—(please God !)—it is enough?

Not in this world of hope deferred,

This world of perishable stuff;

Eye hath not seen, nor ear hath heard,

Nor heart conceived that full "enough":

Here moans the separating sea,

Here harvests fail, here breaks the heart;

There God shall join and no man part,

All one in Christ, so one—(please God !)—with me.

—*Christina Rossetti.*

**Reading.**

The divine purpose of pain in relation to mankind is to purify and to elevate the character of those who rightly endure it. God in His love seeks to develop character in man, and He has in His wisdom made pain an instrument in accomplishing this end. The divine purpose of pain is the formation of a holy character in man, in the face of moral evil. To make men good as He is good, that is the problem. It is a problem which He sees can best be solved by means of suffering. When we say that men learn wisdom by experience, we mostly mean by experience of something painful. Of course, the most obvious form of correction is that in which the suffering can be recognised by the sufferer as merited, because due to his own misdeeds. But apart from such casual connection, what we call unmerited suffering exercises the same influence in an even greater measure. Its forces, not being exhausted in the work of neutralising past evil, are able to expand and expend themselves in a positive direction, elevating, refining, dignifying the character to an infinite degree. The men of sorrows are the men of influence in every walk of life. Martyrdom is the certain road to success in any cause. Even more than knowledge, pain is power. And all this because it develops the latent capacities of our being as no other influence can. It requires no mystic insight to see the truth of this. . . . In the sufferings and death of Christ we see God, clothed in our nature, voluntarily entering into fellowship with all forms of human pain; and thus recognising His responsibility for its permission. And we gain more than this. The cross and passion of Jesus Christ were but the prelude to the resurrection and glorification of the Divine Sufferer, "Who for the joy that was set before Him endured the cross, despising the shame, and hath sat down at the right hand of the throne of God."—*Heb.* xii. 2. (*From The Natural Religion.*)

I lift mine eyes to see: earth vanisheth.
I lift up wistful eyes and bend my knee:
Trembling, bowed down, and face to face with Death,
I lift mine eyes to see.

Lo, what I see is Death that shadows me:
Yet whilst I, seeing, draw a shuddering breath,
Death like a mist grows rare perceptibly.

Beyond the darkness light, beyond the scathe

Healing, beyond the Cross a palm-branch tree,

Beyond Death Life, on evidence of faith:

I lift mine eyes to see. —*Christina Rossetti.*

### Notes and Queries.

**The contents of the Editor's Letter-box have now reached such unwieldy proportions that it has become necessary to establish a hard and fast rule regarding Answers to Correspondents. In future, all questions requiring replies will continue to be answered in this column without any fee. If an answer is required by letter, a fee of half-a-crown must be enclosed with the note containing the enquiry. We are always pleased to help our numerous correspondents to the fullest extent, and we can trust them to sympathise in the overwhelming amount of writing which makes the new rules a necessity.**

**Every communication must be accompanied by the writer's name and address, otherwise it will receive no attention.**

*Appointment.*

(161) Can you kindly give me any information as to what steps a trained and certificated nurse of several years' experience should take to obtain a civil Government appointment abroad (the Colonies preferred)? —*Civilian.*

Write to the Hon. Secretary of the Colonial Nursing Association, Imperial Institute, S.W.

*Completion of Training.*

(162) A nurse with seventeen months' training would like to know the name of a hospital where she could complete her training and gain a three years' certificate. She thinks several hospitals have made an arrangement of this kind, but does not know which they are.—*Nurse M.*

The Matron, the Hospital for Diseases of the Chest, Victoria Park, E., would give you details of the arrangement she has made for this purpose upon application.

*Post.*

(163) Will you kindly tell me what daily paper to take in to get a post as matron or head attendant in a public institution?—*M. P.*

The weekly medical journals—*Lancet*, *British Medical Journal*, and The Hospital—and those devoted to County Council and Poor Law interests are the most frequently used mediums for making such requirements known. Most of the daily papers publish occasional advertisements, but very few in comparison with the weekly ones named.

*Workhouse Infirmaries.*

(164) Will you please advise me as to whether the three years' training and certificate given at a workhouse infirmary is considered as good as that obtained at a general hospital?—*J. T.*

It entirely depends upon the workhouse infirmary. The training in some such infirmaries is superior to that of some general hospitals.

*Training School.*

(165) Will you kindly let me know if the Taunton and Somerset Hospital is considered a good training school? and if its certificate would qualify the holder for a good appointment afterwards?—*Grimey Lotus.*

The Taunton and Somerset Hospital maintains a resident medical officer, and so fulfils the requirements of the Local Government Board as regards the training of superintendent nurses in Poor Law institutions.

*Matron's Studies.*

(166) I am expecting shortly a call to a large London hospital. I should be very glad if you could tell me what books to use for a little preparatory study, which I thought would help me as I am not very quick. I have taken in The Hospital for nearly two years; the medical terms sometimes puzzle me. Is there a dictionary that I could refer to? Do you know of a home or hospital that would be glad of old magazines?—*Mary G.*

1. You do not say on what subjects you want the books. "The Matron's Course," by Miss S. E. Orme, late Lady Superintendent of the Temperance Hospital; and "Hospital Sisters and their Duties," by the Matron of the London, are both useful for training nurses. 2. Honnor Morten's little volume compiled for the use of nurses would probably suit you, "The Nurses' Dictionary of Medical Terms and Nursing Treatment," price 2s., from the Scientific Press, or Hoblyn's Dictionary of Medical Terms. 3. The nearest workhouse infirmary would doubtless welcome any popular literature.

*Swedish Movements and Massage.*

(167) Could you tell me of a teacher who would give a short course of lessons in Swedish movements and massage for curvature?—*F. A. Mashiter.*

Apply to the Secretary, Society of Trained Masseuses, 12, Buckingham Street, Strand, W.C.

*L.O.S.*

(168) Could you tell me where I am to apply for form to fill in necessary number of obstetric cases, as required by L.O.S.?—*M. H.*

Write to the secretary, London Obstetrical Society, 20, Hanover Square, London, W., for full particulars of the examination.

*Advanced Nursing.*

(169) Will you kindly inform me of some book for nurses on advanced surgery, also of one on medical cases? I have several nursing manuals, but require books that describe more fully.—*A. M.*

The Scientific Press has recently published a "Handbook for Nurses," by Dr. Watson, which is very highly spoken of for the purpose you name, price 5s.

*Standard Books of Reference.*

"The Nursing Profession: How and Where to Train." 2s. net.
"The Nurses' Dictionary of Medical Terms." 2s. 6d. net.
"Burdett's Series of Nursing Text-Books." 1s. each.
"A Handbook for Nurses." (Illustrated.) 5s.
"Nursing: Its Theory and Practice." New Edition. 3s. 6d.
"Helps in Sickness and to Health." Fifteenth Thousand. 5s.
All these are published by The Scientific Press, Ltd., and may be obtained through any bookseller or direct from the publishers, 28 & 29, Southampton Street, London, W.C.

### Travel Notes.

By Our Travelling Correspondent.

### XLIII.—WINTER RESORTS (*continued*).

### Palermo.

Sicily has been coming into fashion lately as a winter health resort, and its popularity is not surprising, for the climate is delicious and the scenery very beautiful; but there are two decided drawbacks, first the long journey to reach its delights, and, secondly, the heavy cost thereof. If, however, one is in the comfortable position of ability to say "Money is no object," no place would make a more agreeable winter residence for those to whom a warm climate is a *sina qua non*. I should not think it quite so suitable for pronounced invalids—the journey is too long. More rain falls in Sicily than on the Riviera, but the climate is very equable, and, as should be the rule with all health resorts, it should not be visited without medical advice. There are several ways of reaching Palermo, the quickest and best is viâ Dover, Paris, Turin, and Naples; first-class £12 19s. 9d., second-class £8 £19s. 6d. The steamer, which you take at Naples at 8 p.m., lands you at Palermo at 7.20 the next morning. To avoid a sea voyage it is possible to take the train to Reggio and cross by small steamer, but this is much more expensive, and considerably more tedious. There are one or two very good hotels in Palermo from ten francs a day pension. Catania and Taormina are in some respects more agreeable places of residence than Palermo. My space is exhausted to-day, but I hope to give an article on the Island of Sicily generally ere long. I may conclude by saying that there is no danger from brigands in the frequented parts of the country, but it is not wise to wander far from the beaten tourist tracks.

### Alassio.

It is only quite lately that Alassio has been regarded as a suitable winter resort. From its position, well sheltered from the north and north-west, it suffers but little from the mistral, but it is exposed on the east, and therefore feels more of the odious easterly wind than is at all agreeable. It is considered that cases of feeble circulation and consumption, where the disease has not made much progress, receive benefit at Alassio. The cost of the journey viâ Calais, Paris, and Turin is £7 12s. 6d. first class, and £5 5s. 1d. second; viâ Dieppe £6 7s. 10d. first class, and £4 11s. 1d. second. The best halting places will be, Paris and Turin. The hotel accommodation is still somewhat limited. The best is the Grand, and another very popular house is the Suisse. There is an English church and a circulating library, and what is a much appreciated advantage, an institute for English trained nurses at the Châlet Santa Croce. At this establishment a few resident patients are taken. We gave a short notice of this nursing centre and the town of Alassio in the "Nursing Mirror" of January 14th. It is a nice spot for excursions for those able to take them, and for others, whose locomotive powers are limited, there is a most perfect sandy beach.

### The Bay of St. Malo.

I have lately received several inquiries as to the fitness of Dinard and the Bay of St. Malo generally to be a winter resort, and I think this is a good opportunity to clear up some misapprehension on the subject. Dinard and the neighbouring towns are but little south of England, and cannot be considered as warm places, such as the Riviera; the advantage they have over the south of Devon and Cornwall is that there is less persistent rain and more sun, enabling invalids to be out of doors much more than in England. The climate is somewhat relaxing, and is, therefore (combined with its only comparatively higher temperature), hardly to be recommended for those suffering from incipient consumption. At the same time there are many cases where a further flight south or to the Higher Alps is entirely out of the question, and such cases may bear a winter at Dinard, St. Enogat, or Paramé far better than they would in the soft humidity of South Devon. Moreover, the journey being so short, convenient, and inexpensive, is an immense advantage, enabling the friends of the semi-invalid to run to and fro very frequently. The cost first class, viâ St. Malo, is £1 15s. 10d.; second class, £1 5s. 10d. Arrived at St. Malo the steam ferry takes you over to Dinard for twopence, and if your destination is Paramé or St. Servan a fiacre lands you at your hotel in a quarter of an hour. Dinan, which is some sixteen miles inland, is also a charming place at any season of the year, a most splendid centre for excursions, either on foot, bicycle, or carriage.

### Spezia.

There is great difference of opinion concerning the advantages of a winter residence at Spezia. From personal observation I can testify to its health-giving properties in some cases, especially those of chronic bronchitis, and its sedative air enables those usually unable to sleep well by the seaside to overcome this difficulty most satisfactorily. It has a splendid supply of pure water from a source in the Apennines, a very great advantage I need hardly say. The journey viâ Dover, Paris, and Turin is £7 18s. 8d. first class, and £5 5s. 10d. second; viâ Newhaven and Dieppe £6 6s. 4d. first class, and £4 8s. 4d. second. The hotel accommodation is good and reasonable. During the winter English Church service is held in the principal hotel, the Croce di Malta. Spezia itself is not very beautiful, though lately immensely improved by the substitution of well-planned public gardens in the place of the Government coal yards, &c. . . . now moved to a discreet distance. The excursions are varied and beautiful, and I shall hope to say more about them another day.

### TRAVEL NOTES AND QUERIES.

On the Moselle (Pyramid).—I think the place you mean is Schloss Elz. Whether you can get leave to sketch inside I cannot say; the interior is not generally shown, but I think possibly if you wrote to the steward a week beforehand you might be permitted to sketch. Still it is doubtful. You reach it from Moselkern, where there is a nice little inn. Schloss Elz is four miles up the valley.

Florence for Two (Vanessa).—I think your plan might succeed well, but you would not, of course, embark on it without consulting a doctor about your sister. Florence is not at all warm in the early spring, but it is flooded with sunshine. Rooms on the Lung Arno are very dear, but there are plenty of rooms high up and facing south more in the city. During your hours of study she would find plenty to amuse her in the galleries, museums, and wonderful churches.

Arcachon (Economy).—No, it is not an expensive place, cheap as compared to Biarritz but dearer than St. Jean de Luz. Fare, first-class single, the cheapest route via Dieppe, £4 10s 5d.; second class, £3 3s. 7d. A very good way if you are good sailors is by boat all the way to Bordeaux, first-class, £2 13s. 5d.

### To Nurses.

We invite contributions from any of our readers, and shall be glad to pay for "Notes on News from the Nursing World," or for articles describing nursing experiences, or dealing with any nursing question from an original point of view. The minimum payment for contributions is 5s., but we welcome interesting contributions of a column, or a page, in length. It may be added that notices of entertainments, presentations, and deaths are not paid for, but, of course, we are always glad to receive them. All rejected manuscripts are returned in due course, and all payments for manuscripts used are made as early as possible at the beginning of each quarter.

### OUR CONVALESCENT FUND.

We acknowledge, with many thanks, £1 from A. S. C., Stanley Mount, Oxton.

